# Investigation of the Deformation Dependence of Polymer Films on Various Physical Factors

**DOI:** 10.3390/polym17212853

**Published:** 2025-10-26

**Authors:** Anatoliy I. Kupchishin, Marat N. Niyazov, Sergey A. Ghyngazov

**Affiliations:** 1 Abai Kazakh National Pedagogical University, Dostyk Avenue 13, Almaty 050010, Kazakhstan; ankupchishin@mail.ru; 2 Al-Farabi Kazakh National University, Al-Farabi Avenue 71, Almaty 050040, Kazakhstan; 3 Tomsk Polytechnic University, 30 Lenina Avenue, 634050 Tomsk, Russia

**Keywords:** polymer films, polyethylene, deformation, ion and electron irradiation, mechanical properties, return deformation, deformation models, exponential model, quadratic model, deformation rate

## Abstract

In this work, models of the deformation behavior of polymer films of polyethylene and polyvinyl chloride are developed and analyzed, taking into account the influence of thickness, mechanical stress, temperature, time and dose of electron and ion irradiation. Experimental studies included tensile tests of polyethylene films of different thicknesses irradiated with krypton ions and electrons, as well as measuring the return deformation and its rate. It is shown that the quadratic and exponential models best describe the dependences of deformation on stress. Analytical formulas for the rate and acceleration of deformation are obtained, taking into account the influence of temperature and radiation dose. The results demonstrate a significant increase in the elastic properties and return deformation of irradiated samples, which is explained by the cross-linking of macromolecules and changes in the molecular structure under the influence of radiation. The proposed models and formulas can be effectively used in the development of devices and systems for monitoring the deformation of polymeric materials under radiation exposure in the aerospace, nuclear and electronic industries. Using the statistical analysis method, it was shown that the exponential model describes the dynamics of polyethylene deformation with a determination coefficient R^2^ = 0.985, which significantly exceeds the accuracy of the linear model (R^2^ = 0.85).

## 1. Introduction

In the modern world, polymeric materials are taking an increasingly important place in various areas of industry, science and technology. Polymers, due to their unique properties such as lightness, high strength, chemical resistance and manufacturability, are gradually replacing traditional metal and ceramic materials in the electronics and aerospace industries, as well as in medicine and energy. In this regard, it is especially important to understand the behavior of polymers under extreme operating conditions, including exposure to radiation, which opens up new prospects for the development of highly effective and durable materials. The relevance of such research is due to the increased interest in studying the influence of ion and electron irradiation on the mechanical and structural properties of various materials, including polymer films, which are widely used in various fields of science and technology. Radiation exposure can significantly affect the physical and mechanical characteristics of polymers, causing structural transformations and modifications, which ultimately affect their deformation behavior. In this regard, the study of the deformation processes of polymer films, both in non-irradiated and irradiated states, becomes an important task to ensure the reliability and durability of materials under extreme operating conditions. Over the past three years, a number of studies devoted to the analysis of the effect of irradiation on the properties of various materials have been published in international scientific journals. Thus, in work [1], the effect of successive irradiation with krypton and helium ions on the composition and structure of CoCrFeNi and CoCrFeMnNi alloys was investigated, which makes it possible to understand the mechanisms of structural changes under radiation exposure. In the study [2], polymorphic transformations in ZrO_2_ ceramics under the influence of heavy ions were studied, and the features of anisotropic deformations associated with these transformations were revealed. The work [3] presents a comprehensive analysis of the effect of Xe^23+^ heavy ion irradiation doses on the structural, optical and strength properties of AlN ceramics, demonstrating the relationship between structural distortions and changes in the mechanical characteristics of the material. The studies [4,5] consider the role of pre-existing defects and radiation damage in IG-110 nuclear graphite and CeO_2_ microstructure ceramics, which is of particular importance for the development of materials used in nuclear reactors and other radiation-resistant systems. Particular attention is paid to the study of polymers. In ref. [6], quasi-static and dynamic characteristics of various crystallized and amorphous polymers, including PVB, EVA, TPU and SG, were experimentally investigated at different strain rates. In ref. [7], observations of the nanoscale plasticity of ion-irradiated graphite were carried out using the IN SITU transmission electron microscopy method, which provides a deep understanding of deformation processes at the micro level. The use of discrete element methods for modeling the dependence of deformation on rate in granular materials is presented in [8]. The article [9] evaluates the effect of proton irradiation on the electrical conductivity of polyimide, one of the most widely used polymeric materials. In ref. [10], the dynamic and post-dynamic recrystallization of the TC18 alloy was studied at different strain rates, which allows for a better understanding of the mechanisms of structural changes under mechanical action. In addition, in the works [11], an analysis of the influence of electron and ion irradiation on the mechanical properties of various polymers was carried out with the aim of identifying universal patterns of change in deformation behavior under radiation exposure.

The aim of this work is to develop and analyze models of the deformation behavior of polymer films taking into account various parameters, including irradiation modes, deformation rate and structural features, as well as to determine the characteristic differences in the deformation of non-irradiated and irradiated samples to identify patterns associated with radiation exposure and their influence on the performance characteristics of materials.

The primary novelty of this work lies in the derivation and experimental verification of new, unified nonlinear (quadratic and exponential) models for strain, strain rate, and strain acceleration that account for complex structural changes induced by two irradiation modes (ions and electrons) in polyethylene (PE) and polyvinyl chloride (PVC) film. Furthermore, a systematic analysis of how specimen geometry (width and length) affects the tensile strength and strain characteristics of PVC under these modified conditions represents a novel and practical contribution to engineering knowledge. The proposed models can be used to improve the reliability of critical components in: (1) housings and insulation elements of ionizing radiation detectors in spacecraft; (2) flexible substrates and printed circuit boards in devices operating in reactor cores; (3) protective cable sheaths and insulators in industrial X-ray systems.

## 2. Experimental

The materials selected for the study were commercial low-density PE film with a thickness of 23 µm (PE23) and 100 µm (PE100), as well as plasticized PVC film with a thickness of 100 µm (PVC100). All samples were standard industrial grades used for packaging and, according to the manufacturer’s specifications, did not contain special radiation-resistant or stabilizing additives that could significantly alter the crosslinking/degradation mechanism under irradiation. Using a special cutting device, the PE films were cut into strips 5 mm wide. The working length of the samples was 5, 7, 10, 12 cm. And PVC100 films were cut into strips 3, 5, 7, 10 mm wide and 4, 5, 6, 7, 8 cm long. Film samples of PE23 were irradiated with krypton ions (energy 147 MeV) at different flux densities: 1.5 × 10^6^, 1.6 × 10^7^, 5.0 × 10^8^; and 1.0 × 10^9^ ions/cm^2^. The calculated absorbed doses for these fluxes were D1, D2, etc. (during the exposure time). The specific absorbed energy was determined by the dosimetry system DRG–01t1 and is 0.004 Gy/s. Samples of PE100 were irradiated with high-energy electrons (2 MeV) with a dose of 200 kGy, which corresponds to an average flux density of 0.16 μA/cm^2^. Tensile tests were carried out on a tensile testing machine RU-50 in accordance with the standard ASTM-D882-18 (Standard Test Method for Tensile Properties of Thin Plastic Sheeting. ASTM International: West Conshohocken, PA, USA, 2018, https://www.astm.org/d0882-18.html, accessed on 23 October 2025) [12]. The stroke speed of 16 mm/min at an AC frequency of 10 Hz was controlled by a CHNT inverter. The temperature of the material during the studies was 23 °C.

In order to conduct studies of the dependence of relative elongation (Elongation, ε) on stress (Mechanics stress, σ) and other dependencies, we have modernized the setup, which provides the ability to measure parameters using motion and force sensors under various loads and their change over time. The setup uses an interface with motion and force sensors from Science Cube. The frequency of data collection on deformation was 2.5 mm·s^−1^, and the measurement of mechanical stress is different due to the change in Young’s modulus during elongation. The choice of types of irradiation and materials was determined by several factors. The types of materials (PE and PVC) were chosen due to their widespread use and relevance for the nuclear and aerospace industries. Specific parameters, such as film thickness, were determined by their availability from manufacturers. Ion and electron irradiation were chosen due to the availability of appropriate equipment at the researchers’ disposal—an ion and electron accelerator. This made it possible to conduct complex experiments and comprehensively study how different types of radiation exposure affect the deformation properties of polymers. The choice of different geometric sizes of samples made it possible to establish that these parameters significantly affect the strength and deformation characteristics of materials, which must be taken into account in engineering calculations. All experimental data, including the dependences of strain on stress, time, temperature, and irradiation dose, were obtained from at least five (*n* = 5) independent measurements for each experimental point. This number of repetitions ensured the necessary statistical significance for calculating the standard deviations presented in the paper. Appendix A to the article shows the formulas for the deformation models for thin films.

## 3. Results and Discussion

**Figure 1:**
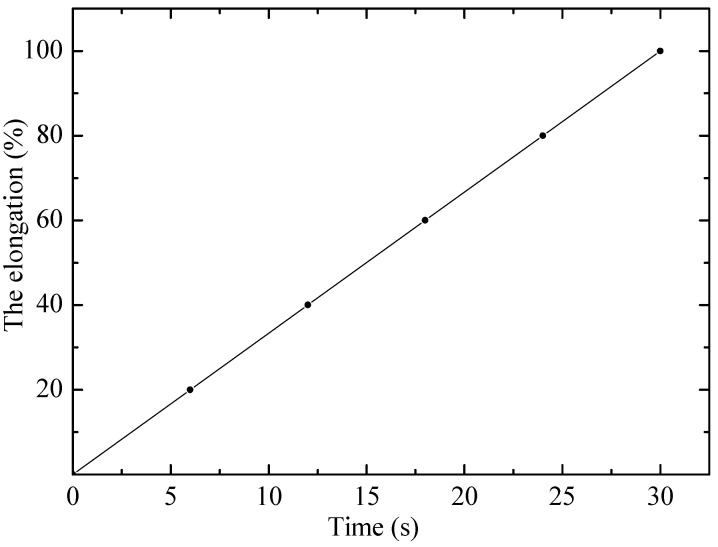
Dependence of relative elongation on time for non-irradiated PE23 sample. Points–experiment, line–linear model.

**Figure 2:**
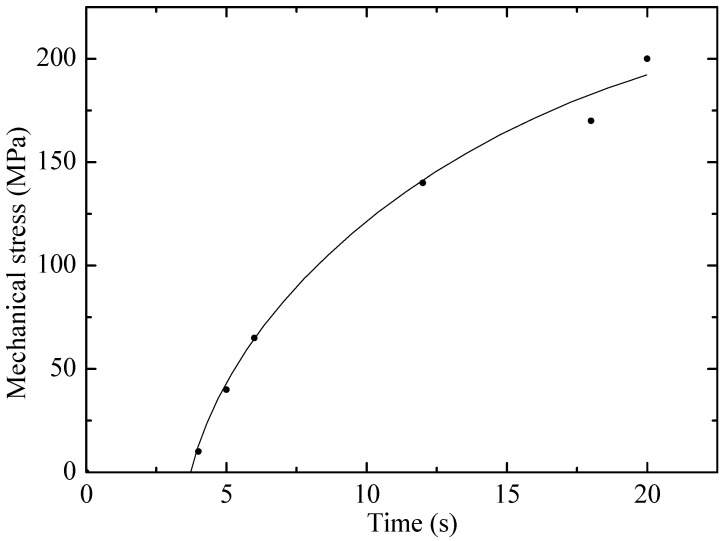
Dependence of mechanical stress on time for non-irradiated PE23 sample. Points–experiment, line–exponential model.

The results of tensile tests of irradiated and non-irradiated PE samples are shown in Figure 1 and Figure 2 as graphs of deformation versus time and mechanical stress versus time, where the experiments (points) are compared with various calculation models (lines) proposed by the authors. Visual analysis confirms that the linear (1) and exponential (4) models describe the experimental results with a high degree of convergence, which indicates their applicability.

The results show that the proposed models can be used for analytical description of the behavior of materials and prediction of their properties under further stretching. The obtained models can serve as a basis for numerical modeling of material behavior under complex loading conditions, for example, in finite element calculations. Thus, the formulas have high practical significance for engineering calculations and materials science.

Figure 2 shows that with increasing stress, the deformation in the elastic region of materials first increases linearly, which is described by Hooke’s law, and then sharply increases according to the exponential law. That is, in general, the entire dynamics of PE stretching is not explained by Hooke’s law. The authors of [13] propose to represent this dependence as an exponential. In the initial phase of stretching, the material demonstrates linear deformation, which corresponds to elastic behavior according to Hooke’s law. However, as the stress increases, the linear section passes into the region of nonlinear deformation growth, indicating the onset of plastic flow. It is in this nonlinear regime that the traditional Hooke’s law ceases to be applicable, since it does not take into account irreversible changes in the structure of the material. The exponential model proposed in [14] describes this phase well, reflecting the strengthening and viscoelastic behavior of PE under stretching. This confirms the position that a full understanding of the dynamics of polymer deformation requires a model that takes into account its complex nature, and not just simple elasticity. The deviation from Hooke’s law is a key indication that microstructural changes, such as polymer chain slippage, occur during testing. Thus, using only a linear approach would significantly underestimate the true deformation capabilities of the material. The success of the exponential model makes it a reliable tool for predicting the behavior of polyethylene under conditions where it undergoes irreversible deformation.

The visual analysis presented in Figure 1 and Figure 2 is supplemented by a detailed statistical assessment of the model fit. As follows from the regression analysis, the determination coefficient (R^2^) for the exponential model (5) is R^2^ = 0.985, which demonstrates excellent agreement with the experimental data. This significantly exceeds the value R^2^ = 0.85 obtained for the linear model (2). Such a low determination coefficient of Hooke’s law quantitatively confirms its statistical inapplicability for describing the entire stretching dynamics of polyethylene. The stability of the nonlinear models is also confirmed by the low standard deviation (SD) of their key parameters: for the exponential model, the parameter was β = 0.15 ± 0.01, and for the quadratic model (3), the parameter α was 0.005 ± 0.0002, obtained during the regression analysis. Despite the high prediction accuracy in the operating range, the limitations of the exponential model in terms of applicability should be noted. The model is not designed to accurately describe the material’s behavior under extreme conditions beyond its operational stability. In particular, at critical temperatures (above 60 °C for polyethylene), the model loses accuracy, as deformation becomes determined by phase transitions and thermal degradation rather than viscoelastic processes. Similarly, at excessively high irradiation doses, when chain scission completely dominates crosslinking, leading to material degradation, the proposed exponential relationships describing hardening become invalid. Therefore, the model remains valid within limits where molecular changes lead to controlled modification of properties rather than their complete destruction.

**Figure 3:**
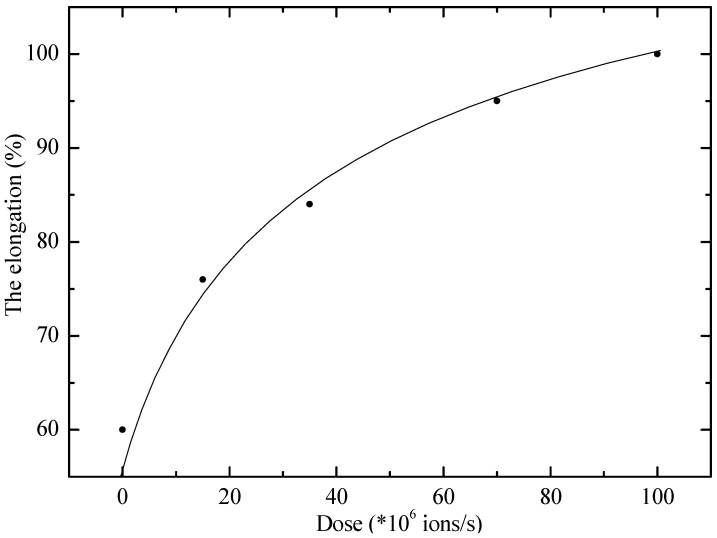
Dependence of the relative elongation of the PE sample on the irradiation dose with ions with an energy of 147 MeV. Points–experiment; line–calculation according to the model.

Figure 3 shows the dependence of relative elongation on the irradiation dose of 147 MeV ions for a PE sample. From its examination it is evident that with increasing dose the deformation gradually increases along an exponential curve and then reaches saturation. At a dose of 10^8^ ions/s, the maximum elongation of the sample increases approximately 1.7 times compared to the ultimate deformation of the control samples of the present experiment (with a standard deviation of SD = 0.08).

The results obtained are consistent with the data of a number of studies in which radiation exposure led to modification of the molecular structure of polymers, including the formation of cross-links (cross-linking) between macromolecules, and are explained by the formula $\varepsilon =\;\varepsilon_{0}\times (1-\exp\;\left({-D/D}_{0}\right))$. Such structural changes, as a rule, contribute to an increase in elasticity and resistance to plastic deformations due to the limitation of chain mobility [14].

The molecular mechanism underlying the observed increase in elastic properties and strain is due to radiation-induced chemical reactions. Energy absorption by ions or electrons leads to ionization and excitation of polymer chain atoms, causing homolytic cleavage of C-H bonds and, consequently, the formation of free radicals (macroradicals) along the chain. In PE under ambient conditions, the primary process dominating at these doses is cross-linking: two adjacent macroradicals on different chains recombine, forming a new covalent C-C bond between the chains. This leads to the formation of a three-dimensional molecular network that limits the mobility of individual chains and increases resistance to plastic deformation, thereby increasing elasticity and tensile strength until saturation is reached at high doses. The competing process is destruction (breakage of the C-C main chain) which is dominant at higher doses or in the presence of oxygen, which explains the subsequent decrease in deformation characteristics as evidenced by data on samples irradiated to a dose of 200 kGy.

It is important to note that results may vary under real industrial conditions, due to the heterogeneity of the composition of commercial polymers. Although the studies were conducted on standard grades of polyethylene and PVC without special radiation additives, most commercial films contain antioxidants, stabilizers, and plasticizers. The presence of such additives can significantly influence radiation-induced chemical processes: antioxidants can reduce cross-linking efficiency by scavenging free radicals, resulting in smaller changes in strength and elasticity than predicted by the pure model. Conversely, plasticizers (especially in PVC) can accelerate the loss of mechanical integrity during irradiation by promoting the migration and degradation of molecular fragments. Therefore, for accurate engineering predictions, it is necessary to take these factors into account when using models as a baseline for the pure polymer.

The increase in deformation of irradiated samples may be due to an increase in cohesion in the molecular network of the material and an increase in elastic restorative forces. Such an effect has practical significance for increasing the durability of polymer products used under radiation exposure conditions.

**Figure 4:**
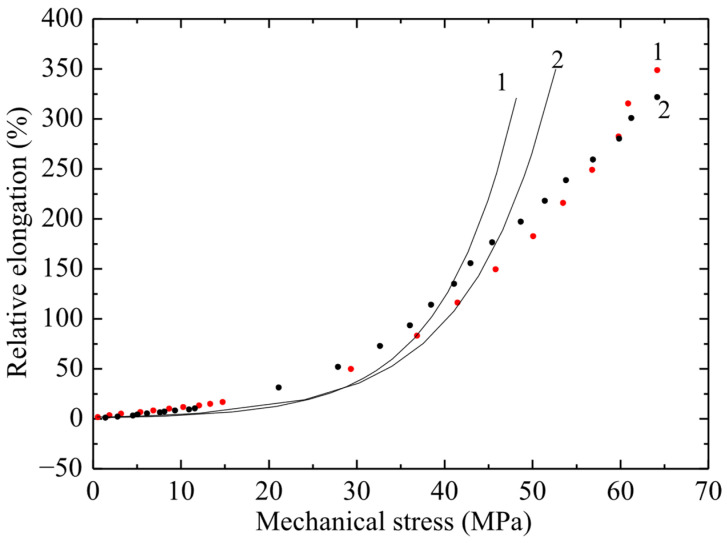
Dependence of relative elongation on engineering mechanical stress of PVC film 3 mm wide and working length 50 mm (curve 1) and 80 mm (curve 2). Points–experiment; line–calculation using the exponential model.

**Figure 5:**
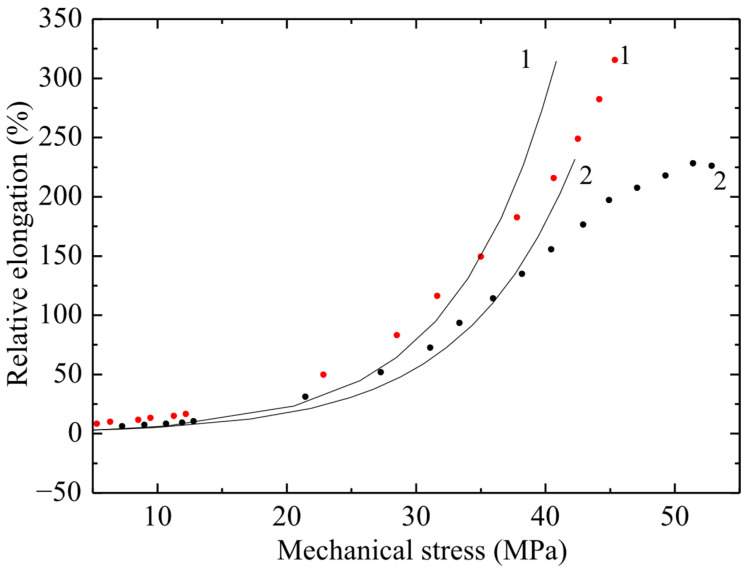
Dependence of relative elongation on engineering mechanical stress of PVC film 5 mm wide and working length 50 mm (curve 1) and 80 mm (curve 2). Points–experiment; line–calculation using the exponential model.

**Figure 6:**
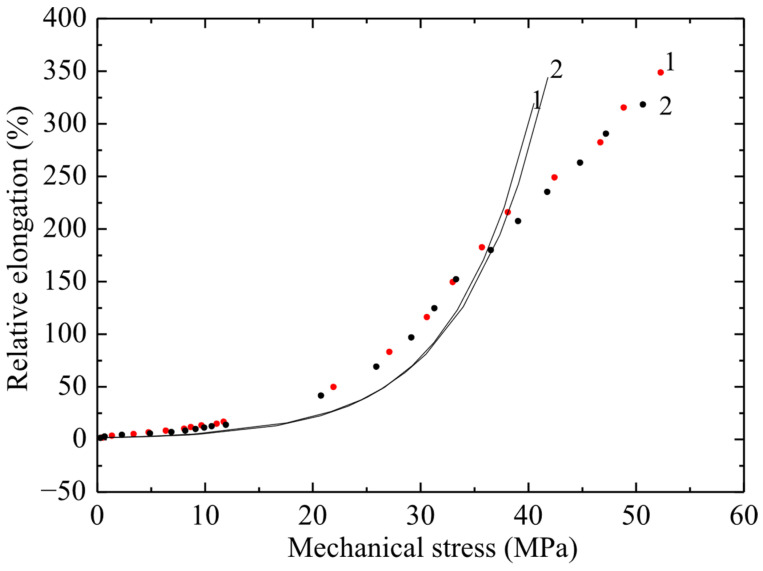
Dependence of relative elongation on engineering mechanical stress for a PVC100 sample with a width of 10 mm and a working length of 50 mm (curve 1) and 80 mm (curve 2). Points–experiment; line–calculation using the exponential model.

The results of tensile tests of non-irradiated PVC samples are shown in Figure 4, Figure 5 and Figure 6 in the form of graphs of the dependence of deformation on mechanical stress. The ultimate strength of the non-irradiated sample according to GOST was 50–65 MPa. Figure 4, Figure 5 and Figure 6 show the dependences of relative elongation on mechanical stress of PVC100 samples with a width of 3, 5 and 10 mm and a working length of 5 and 8 cm. The dots indicate experimental data, and the lines indicate calculations using the exponential model proposed by the authors:

$$\varepsilon \;=\;\varepsilon_{0}(\exp(\;\sigma /\sigma_{0})\;–1).$$

*ε*_0_—the initial value of relative elongation, *σ*_0_—the mechanical stress at which the parameter log(*ε*/*ε*_0_) + 1 decreases by e times [15,16].

The *σ*(*ε*) relationship has no physical meaning, since mechanical stress is an argument, while relative elongation is a function. Therefore, graphs of strain versus mechanical stress are presented. From Figure 5 it follows that there is a significant difference in the maximum deformation of samples 5 and 8 cm long, and it reaches about 100%.

From the obtained results (Figure 4, Figure 5 and Figure 6) it follows that, with increasing stress, the deformation of the material increases exponentially. At the same time, a significant increase in relative elongation is observed with increasing mechanical stress. According to the graphs showing the experimental data, it is evident that the difference in relative elongation of the samples is approximately 50%. When comparing the deformation and mechanical stress readings of samples of different widths, significant differences in deformation characteristics were obtained. PVC samples with a width of 3 mm turned out to be stronger by 5 ± 0.5 MPa compared to films of the same substance with a width of 5 and 10 mm (with a confidence interval of 95%).

The observed relationship, where narrower specimens exhibit increased strength, is explained by the size effect and defect statistics. As the specimen width—that is, the volume of material subjected to engineering stress—increases, the statistical probability of critical microstructural defects, such as microcracks, voids, or processing-induced inhomogeneities, becoming incorporated into this volume increases. These defects act as stress concentrators, and consequently, the specimen fails at a lower average stress. Conversely, narrow specimens (3 mm) have a statistically lower number of critical defects within the working volume, resulting in higher measured tensile strength. This effect is typical for brittle or quasi-brittle materials, such as PVC, under these testing conditions.

**Figure 7:**
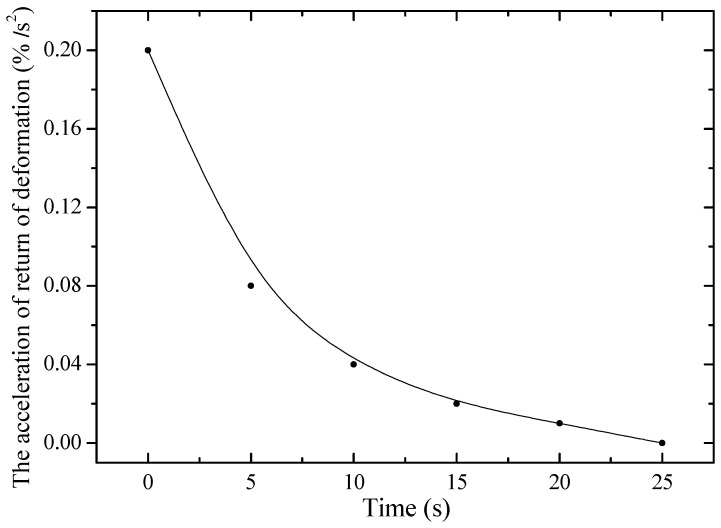
Dependence of acceleration of return deformation on time of non-irradiated PE23 5 cm long under static stress of 80 MPa.

Figure 7 shows the dependence of the acceleration of return deformation on time for unirradiated PE. It is evident that at a constant static stress of 80 MPa, the acceleration decreases with time, reaching a value of about 0.2%/s^2^. This behavior is associated with the straightening of randomly located polymer chains at the initial moment of deformation. After this, the acceleration begins to decrease, which is due to the resistance of the material to further deformation. This resistance increases due to a decrease in the cross-sectional area, which, in turn, leads to an increase in the rigidity of the sample.

**Figure 8:**
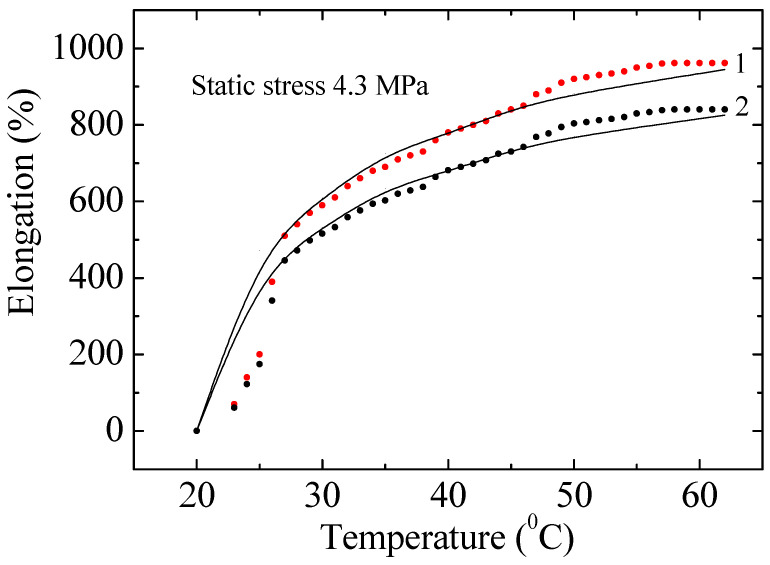
Experimental and calculated data on the dependence of the relative elongation of non-irradiated (1) and irradiated with a dose of 200 kGy (2) PE100 on temperature at a static stress of 4.3 MPa. Points–experimental data; lines–calculations. Vertical error bars represent the standard deviation (±SD) of *n* = 5 measurements.

Figure 8 shows the experimental and calculated data on the dependence of relative elongation on temperature of a non-irradiated and irradiated with a dose of 200 kGy PE100 sample at a static stress of 4.3 MPa. It is evident that PE does not belong to heat-resistant polymers and breaks at 60 °C. All presented experimental data, including those in Figure 8, were obtained from at least five independent measurements (*n* = 5). Error bars in the graphs represent the standard deviation (±SD), confirming the statistical stability of the results. The experimental data can be described within the framework of the exponential (cascade-probability) model (Formula (A20), Appendix A). As follows from Figure 8, the calculated dependencies satisfactorily describe the obtained experimental curves. After irradiation, the deformation properties of PE change significantly. For example, the relative elongation of PE irradiated with a dose of 200 kGy decreased by 12.5 ± 1.5% compared to the deformation of the unirradiated sample. The reason for this is the nanoscale destruction and degradation of chains in the polymer structure. In Figure 7, the curves for doses of 0, 10, 30, 50, 70 and 200 kGy are very close to each other, so only the two extreme ones are shown—0 and 200 kGy. The experimental curves for samples with doses of 10–100 kGy lie between the curves with irradiation doses of 0 and 200 kGy, which indicates the observation of a pattern.

**Figure 9:**
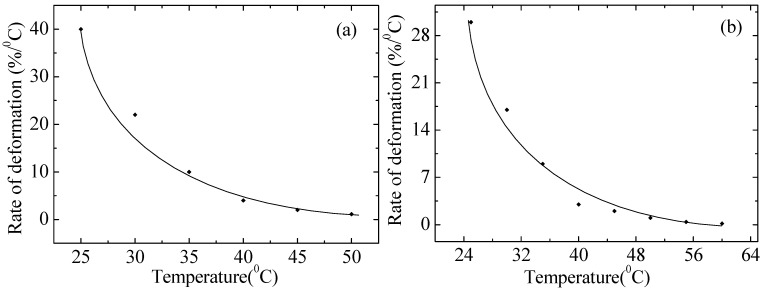
Dependence of the strain rate on the temperature of non-irradiated (**a**) and irradiated with a dose of 200 kGy (**b**) PE100 at a static stress of 4.3 MPa. Points–experiment; lines–calculations according to the model.

Figure 9 shows the dependence of the strain rate on the temperature for non-irradiated and irradiated PE. It is evident that, with increasing temperature, the strain rate of the polymer drops drastically. PE is a polymer that is not resistant to temperature effects and its melting point is about 110 °C, while at 60 °C it is completely stretched and stops elongating any more, as evidenced by the graphs, and after several minutes it breaks at the same strain value. At temperatures above a certain value (usually about 50–130 °C for ordinary polyethylene), the molecules begin to vibrate and lose order, which leads to melting of the material. Additionally, in PE, the main types of bonds between molecules are van der Waals forces—relatively weak interactions that do not provide high heat resistance. The rate of return strain decreased after irradiation. A possible reason is the degradation of chains due to the destruction of macromolecules and oxidation reactions. The experimental data are in good agreement with Formula (A21) in Appendix A.

An analysis of the thermomechanical behavior of polyethylene (Figure 8 and Figure 9) reveals a sharp decrease in mechanical stability with increasing temperature. For low-density PE, the grade used in this study, the glass transition temperature typically ranges from −120 to −80 °C. Therefore, all experiments were conducted in the highly elastic state (above the glass transition temperature). However, the critical loss of strength at 60 °C is due to approaching the melting point of the crystalline phase, which for PE is approximately 105–115 °C. We intentionally stopped the experiment at 60 °C as, at this temperature, the material under static loading (4.3 MPa) enters a state of rapid creep and failure, which is beyond the range of stable performance characteristics. The observed sharp drop in strain rate in this range, especially for irradiated samples, emphasizes that radiation modification does not improve, but may even reduce, the thermal stability of the material near critical temperatures.

## 4. Conclusions

The conducted studies made it possible to develop and verify a set of analytical models that adequately describe the deformation behavior of thin polymer films (PE and PVC) in a wide range of external influences.

The main conclusions confirmed by the results are as follows.

1. Limitations of classical models. The main innovation lies in the development and verification of quadratic and exponential models. In contrast to the linear approach, which is statistically inapplicable to the full dynamics of deformations, the models used demonstrate the highest predictive accuracy (for example, the coefficient of determination R^2^ for the exponential model reaches 0.985), which is a significant improvement compared to the data described by Hooke’s law (where R^2^ is 0.85).

2. Effect of radiation modification. Radiation irradiation (with ions and electrons) is an effective method for modifying polymers. Increasing the irradiation dose leads to increased strain up to saturation due to the dominance of radiation-induced crosslinking of macromolecules and, consequently, an increase in their elastic properties.

3. The reverse effect of irradiation on recovery. Irradiation with krypton ions reduces the rate of return strain in polyethylene, indicating a change in the molecular structure, including deterioration of polymer chain slip and an increase in their rigidity.

4. Decreased thermal stability. Irradiation reduces the thermal stability of polyethylene, which is manifested by a sharp drop in strength and a catastrophic decrease in strain rate with increasing temperature.

5. Effect of geometric parameters. The deformation characteristics of films depend on their geometric dimensions. It has been established that longer samples exhibit higher ultimate strain, while narrower samples exhibit increased strength. The developed analytical models and the resulting quantitative relationships can be used for predictive calculations and targeted control of the properties of polymer structural components operating under radiation and thermomechanical stress.

These results open up several avenues for further work. Key areas include the following.

1. Expanding the range of materials. Developing and verifying analytical models for amorphous polymers, such as PMMA, and crystalline polymers, such as PTFE, to create a universal prediction tool.

2. Multiphysics modeling. This involves integrating developed analytical formulas into numerical methods (e.g., the finite element method—FEM) to create full-scale multiphysics models capable of predicting not only mechanical but also thermal/radiation degradation of complex structural components.

3. Environmental Impact. A systematic study of the relationship between combined radiation exposure and other critical factors such as humidity and cyclic mechanical loading to obtain more comprehensive data on the durability of materials under real-world conditions.

## Data Availability

The original contributions presented in this study are included in the article. Further inquiries can be directed to the corresponding author.

## Appendix A

Deformation models for thin polymer films
1. Linear model. The simplest differential equation is written for it:

(A1) $$dl=\alpha_{1}\times d\sigma$$

where $\alpha_{1}$ is a constant characterizing the properties of the material.

Integrating from *l*_0_ to *l*_0_ + Δ*l* and from zero to σ, and introducing the notation $\Delta l/l_{0}=\varepsilon$ we obtain:

(A2) $$\varepsilon =\frac{\varepsilon_{0}}{\alpha_{1}}\times \sigma$$

The modulus of elasticity or Young’s modulus can be defined as E = l0/*α*1.

2. Quadratic model. Similarly to (A1), we will compose a differential equation for the quadratic model: $dl=\alpha_{2}\cdot \sigma d\sigma$. Integrating over l and σ, in the same limits as above, we ultimately obtain:

(A3)
$$\varepsilon =\alpha_{2}\times \sigma^{2}$$

3. Exponential model. The differential equation is represented as: dl=l⋅dσ. By integrating, we obtain the formula for this model:

(A4)
$$\varepsilon =\;\exp\;\left({\sigma /\sigma }_{0}\right)-1$$

For quasi-stationary parts, Formula (A4) will take the form:

(A5) $$\varepsilon =\;\varepsilon_{1}+\exp\;\left({-\sigma^{|}/\sigma }_{0}\right)-1$$

Differentiating Formula (A2) with respect to σ, we obtain:

(A6) $$d\varepsilon /d\sigma =l_{0}/\alpha_{1}$$

The strain rate for model 2. Similarly to the linear model, we differentiate by σ and obtain:

(A7) $$d\varepsilon /d\sigma =2\alpha_{2}\times \sigma$$

Strain rate for the exponential model. The differential equation is written as:

(A8) $$\frac{d\varepsilon }{d\sigma }=\left(\frac{1}{\sigma_{0}}\right)\times \exp\left(\frac{\sigma }{\sigma_{0}}\right)$$

Acceleration of deformation for the linear model:

(A9) $$a=d_{2}\varepsilon /d\sigma^{2}$$

Acceleration for the quadratic model:

(A10) $$a=2\alpha_{2}.$$

Acceleration for the exponential model:

(A11)
$$a=(1/\sigma_{0}^{2})\times \exp(\frac{\sigma }{\sigma_{0}})$$

Deformation model for time dependence:

(A12) $$\varepsilon =\;1-\exp\;\left({-\tau /\tau }_{0}\right)$$

Deformation rate for time dependence. Carrying out similar procedures, we obtain:

(A13) $$\frac{d\varepsilon }{d\tau }=\left(\frac{1}{\tau_{0}}\right)\times \exp\left(\frac{\tau }{\tau_{0}}\right)$$

Acceleration for deformation depending on time:

(A14) $$a=\left(-\frac{1}{\tau_{0}^{2}}\right)\times \exp\left(\frac{\tau }{\tau_{0}}\right)$$

Model of dependence of deformation on radiation dose. Using the same procedure, we obtain:

(A15) $$\varepsilon =\;\varepsilon_{0}\times \exp\;\left({-D/D}_{0}\right),\;и\;\sigma =\;\sigma_{0}\times \exp\;\left({-D/D}_{0}\right)$$

For the deformation rate depending on the dose, we obtain:

(A16) $$\frac{d\varepsilon }{dD}=-\left(\frac{\varepsilon_{0}}{D_{0}}\right)\times \exp\left(-\frac{D}{D_{0}}\right)$$

and the following:

(A17) $$\frac{d\sigma }{dD}=\left(\frac{\sigma_{0}}{D_{0}}\right)\times \exp\left(-\frac{D}{D_{0}}\right)$$

Model of return deformation for time dependence:

(A18) $$\varepsilon_{r}=\;1-\exp\;\left({\tau /\tau }_{0}\right)$$

The rate of return deformation for time dependence. Carrying out similar procedures, we obtain:

(A19) $$\frac{d\varepsilon_{r}}{d\tau }=\left(-\frac{1}{\tau_{0}}\right)\times \exp\left(-\frac{\tau }{\tau_{0}}\right)$$

Acceleration for return deformation depending on time:

(A20) $$a_{r}=\left(-\frac{1}{\tau_{0}^{2}}\right)\times \exp\left(-\frac{\tau }{\tau_{0}}\right)$$

Model of return deformation for temperature dependence:

(A21) $$\varepsilon_{r}=\;1-\exp\;\left(-{t/t}_{0}\right)$$

The rate of return deformation for temperature dependence. Carrying out similar procedures, we obtain:

(A22) $$\frac{d\varepsilon_{r}}{dt}=\left(-\frac{1}{t_{0}}\right)\times \exp\left(-\frac{t}{t_{0}}\right)$$

Acceleration for return deformation depending on temperature:

(A23) $$a_{r}=\left(-\frac{1}{t_{0}^{2}}\right)\times \exp\left(-\frac{t}{t_{0}}\right)$$
